# Corrigendum: Racial and Ethnic Inequities in Mortality During Hospitalization for Traumatic Brain Injury: A Call to Action

**DOI:** 10.3389/fsurg.2022.838428

**Published:** 2022-01-24

**Authors:** Emma A. Richie, Joseph G. Nugent, Ahmed M. Raslan

**Affiliations:** Department of Neurological Surgery, Oregon Health and Science University, Portland, OR, United States

**Keywords:** mortality, disparities, inequities, TBI, neurotrauma, race

There was an error in Table 1B as published in the original article. Secondary payor, discharge disposition, probability of survival, and deaths were overwritten for the propensity-matched cohort with instead a repetition of the demographic characteristics. The corrected and complete version of Table 1B appears below.

**Table 1B:** Patient characteristics stratified by minority status following propensity matching.

		**Total**	**Non-minority**	**Minority**	***p*-value**	**Absolute standardized difference**
*n*		3,000	1,500	1,500		
Age (years)^*^		43.1 (1.65)	43.54 (1.65)	42.66 (1.65)	0.265	0.041
**Categorical age**	15–24	568 (18.9)	275 (18.3)	293 (19.5)	NA	0.120
	25–34	494 (16.5)	249 (16.6)	245 (16.3)		
	35–44	383 (12.8)	174 (11.6)	209 (13.9)		
	45–54	396 (13.2)	201 (13.4)	195 (13.0)		
	55–64	390 (13.0)	210 (14.0)	180 (12.0)		
	65–74	293 (9.8)	157 (10.5)	136 (9.1)		
	75–84	285 (9.5)	131 (8.7)	154 (10.3)		
	85+	191 (6.4)	103 (6.9)	88 (5.9)		
**Sex**	Male	2,172 (72.4)	1,087 (72.5)	1,085 (72.3)	0.967	0.003
	Female	828 (27.6)	413 (27.5)	415 (27.7)		
**Race**	White	1,518 (67.6)	1,500 (100.0)	18 (2.4)	NA	9.000
	Black or African American	99 (4.4)	0 (0.0)	99 (13.3)		
	American Indian or Alaskan Native	59 (2.6)	0 (0.0)	59 (7.9)		
	Asian	142 (6.3)	0 (0.0)	142 (19.0)		
	Hispanic or Latino	346 (15.4)	0 (0.0)	346 (46.3)		
	Pacific Islander or Native Hawaiian	15 (0.7)	0 (0.0)	15 (2.0)		
	Other	68 (3.0)	0 (0.0)	68 (9.1)		
**Ethnicity**	Not Hispanic or Latino	2,624 (87.5)	1,500 (100.0)	771 (51.4)	<0.001	1.375
	Hispanic or Latino	375 (12.5)	0 (0.0)	729 (48.6)		
BMI^*^		26.57 (1.23)	26.87 (1.23)	26.31 (1.24)	0.108	0.099
**Injury type**	Blunt	2,853 (95.1)	1,432 (95.5)	1,421 (94.7)	0.398	0.034
	Penetrating	147 (4.9)	68 (4.5)	79 (5.3)		
**Mechanism of injury**	Fall	1,198 (39.9)	608 (40.5)	590 (39.3)	NA	0.051
	Firearm	104 (3.5)	52 (3.5)	52 (3.5)		
	Motor vehicle traffic—Motorcyclist	87 (2.9)	42 (2.8)	45 (3.0)		
	Motor vehicle traffic—Occupant	616 (20.5)	303 (20.2)	313 (20.9)		
	Motor vehicle traffic—Pedestrian	253 (8.4)	128 (8.5)	125 (8.3)		
	Pedal cyclist, other	91 (3.0)	44 (2.9)	47 (3.1)		
	Struck by, against	341 (11.4)	175 (11.7)	166 (11.1)		
	Transport, other	85 (2.8)	38 (2.5)	47 (3.1)		
	Other	225 (7.5)	110 (7.3)	115 (7.7)		
**Work relatedness of injury**	Work related	129 (4.3)	59 (3.9)	70 (4.7)	0.368	0.036
	Not work related	2,870 (95.7)	1,440 (96.1)	1,430 (95.3)		
GCS on ED presentation^*^		10.62 (1.81)	10.81 (1.8)	10.43 (1.83)	0.142	0.059
ISS^*^		19.8 (1.6)	19.52 (1.6)	20.08 (1.6)	0.101	0.060
**AIS H&N**	3	1,239 (41.3)	623 (41.5)	616 (41.1)	0.220	0.077
	4	1,104 (36.8)	566 (37.7)	538 (35.9)		
	5	646 (21.5)	308 (20.5)	338 (22.5)		
	6	11 (0.4)	3 (0.2)	8 (0.5)		
**TBI severity**	Mild	1,780 (72.6)	912 (74.1)	868 (71.0)	0.219	0.070
	Moderate	169 (6.9)	81 (6.6)	88 (7.2)		
	Severe	503 (20.5)	237 (19.3)	266 (21.8)		
**Transport mode**	Ambulance	2,312 (77.2)	1,147 (76.7)	1,165 (77.7)	0.796	0.073
	Privately owned vehicle	37 (1.2)	17 (1.1)	20 (1.3)		
	Walk in	2 (0.1)	2 (0.1)	0 (0.0)		
	Helicopter	419 (14.0)	213 (14.2)	206 (13.7)		
	Police or law enforcement	1 (0.0)	1 (0.1)	0 (0.0)		
	Auto launch helicopter	0 (0.0)	0 (0.0)	0 (0.0)		
	Other	12 (0.4)	7 (0.5)	5 (0.3)		
	Fixed wing aircraft	211 (7.0)	108 (7.2)	103 (6.9)		
**Advanced directives**	Advance directive, directive to physicians, or living will	62 (2.1)	33 (2.2)	29 (2.0)	0.492	0.058
	Both advance directive AND POLST prior to injury	13 (0.4)	4 (0.3)	9 (0.6)		
	No, neither advance directive nor POLST prior to injury	2,805 (94.6)	1,416 (94.8)	1,389 (94.4)		
	POLST in effect prior to injury	86 (2.9)	41 (2.7)	45 (3.1)		
**Operative status**	Operative patient	1,039 (34.6)	522 (34.8)	517 (34.5)	0.848	0.007
	Non-operative patient	1,960 (65.4)	977 (65.2)	983 (65.5)		
Time to first OR visit (min)^*^		871.31 (5.81)	1012.32 (5.37)	735.10 (6.23)	0.005	0.183
Length of hospital stay (days)^*^		3.8 (3.83)	4.26 (3.4)	3.38 (4.24)	<0.001	0.172
Length of ICU stay (days)^*^		2.15 (3.08)	2.34 (2.92)	1.98 (3.24)	<0.001	0.150
Total hospital charges^*^		46,051.76 (2.73)	47,165.17 (2.71)	44,964.63 (2.75)	0.193	0.048
**Primary payor**	Vehicle insurance	792 (26.4)	398 (26.5)	394 (26.3)	NA	0.090
	Charity	0 (0.0)	0 (0.0)	0 (0.0)		
	Commercial insurance	589 (19.6)	287 (19.1)	302 (20.1)		
	Medicaid	489 (16.3)	251 (16.7)	238 (15.9)		
	Medicare	530 (17.7)	278 (18.5)	252 (16.8)		
	Other health insurance (VA)	127 (4.2)	53 (3.5)	74 (4.9)		
	Self pay	334 (11.1)	167 (11.1)	167 (11.1)		
	Ward of federal government	17 (0.6)	9 (0.6)	8 (0.5)		
	Workman's compensation	122 (4.1)	57 (3.8)	65 (4.3)		
***n***		3,000	1,500	1,500		
**Age (years)**		48.41 (22.12)	48.87 (22.13)	47.96 (22.10)	0.259	0.041
**Categorical age**	15–24	568 (18.9)	275 (18.3)	293 (19.5)	NA	0.120
	25–34	494 (16.5)	249 (16.6)	245 (16.3)		
	35–44	383 (12.8)	174 (11.6)	209 (13.9)		
	45–54	396 (13.2)	201 (13.4)	195 (13.0)		
	55–64	390 (13.0)	210 (14.0)	180 (12.0)		
	65–74	293 (9.8)	157 (10.5)	136 (9.1)		
	75–84	285 (9.5)	131 (8.7)	154 (10.3)		
	85+	191 (6.4)	103 (6.9)	88 (5.9)		
**Sex**	Male	2,172 (72.4)	1,087 (72.5)	1,085 (72.3)	0.967	0.003
	Female	828 (27.6)	413 (27.5)	415 (27.7)		
**Race**	White	1,518 (67.6)	1,500 (100.0)	18 (2.4)	NA	9.000
	Black or African American	99 (4.4)	0 (0.0)	99 (13.3)		
	American Indian or Alaskan Native	59 (2.6)	0 (0.0)	59 (7.9)		
	Asian	142 (6.3)	0 (0.0)	142 (19.0)		
	Hispanic or Latino	346 (15.4)	0 (0.0)	346 (46.3)		
	Pacific Islander or Native Hawaiian	15 (0.7)	0 (0.0)	15 (2.0)		
	Other	68 (3.0)	0 (0.0)	68 (9.1)		
**Ethnicity**	Not Hispanic or Latino	2,624 (87.5)	1,500 (100.0)	771 (51.4)	<0.001	1.375
	Hispanic or Latino	375 (12.5)	0 (0.0)	729 (48.6)		
**BMI**		27.18 (5.91)	27.47 (5.96)	26.91 (5.85)	0.123	0.095
**Injury type**	Blunt	2,853 (95.1)	1,432 (95.5)	1,421 (94.7)	0.398	0.034
	Penetrating	147 (4.9)	68 (4.5)	79 (5.3)		
**Mechanism of injury**	Fall	1,198 (39.9)	608 (40.5)	590 (39.3)	NA	0.051
	Firearm	104 (3.5)	52 (3.5)	52 (3.5)		

*The values displayed are computed from the geometric mean geometric standard deviation.*

*BMI, Body Mass Index; GCS, Glasgow Coma Scale; ISS, Injury Severity Score; AIS H&N, Abbreviated Injury Score of the Head & Neck; TBI, Traumatic Brain Injury; POLST, Physicians Orders For Life-Sustaining Treatment; ICU, Intensive Care Unit; VA, Veterans Affairs*.

## Publisher's Note

All claims expressed in this article are solely those of the authors and do not necessarily represent those of their affiliated organizations, or those of the publisher, the editors and the reviewers. Any product that may be evaluated in this article, or claim that may be made by its manufacturer, is not guaranteed or endorsed by the publisher.

