# Supplementary material for: Racial and Ethnic Inequities in Mortality During Hospitalization for Traumatic Brain Injury: A Call to Action
Source: Front Surg. 2021 Jun 2;8:690971. doi: 10.3389/fsurg.2021.690971 (PMC8207515; doi:10.3389/fsurg.2021.690971)
Supplement: Supplementary file 1 [file Data_Sheet_1.pdf]

Geometric means for continuous variables in unadjusted cohorts stratified by minority status.

	Total	Non-Minority	Minority	p-value	Absolute Standardized Difference
**n**	6,352	4,848	1,504		
**Age***	47.89 (1.63)	49.66 (1.61)	42.61 (1.65)	<0.001	0.313
**BMI***	26.64 (1.23)	26.74 (1.23)	26.3 (1.23)	0.097	0.080
**GCS on ED Presentation***	11.12 (1.75)	11.33 (1.72)	10.44 (1.83)	<0.001	0.142
**ISS (Injury Severity Score)***	19.36 (1.58)	19.14 (1.58)	20.07 (1.6)	<0.001	0.102
**Length of Hospital Stay (days)***	3.85 (3.53)	4.01 (3.3)	3.37 (4.27)	<0.001	0.131
**Length of ICU Stay (days)***	2.12 (2.92)	2.16 (2.82)	1.98 (3.24)	0.007	0.082
**Total Hospital Charges***	44,689.2 (2.66)	44,613.48 (2.63)	44,934.16 (2.75)	0.804	0.007

* The values displayed are computed from the geometric mean

Geometric means for continuous variables in propensity-matched cohorts stratified by minority status.

	Total	Non-Minority	Minority	p-value	Absolute Standardized Difference
**n**	3,000	1,500	1,500		
**Age***	43.1 (1.65)	43.54 (1.65)	42.66 (1.65)	0.265	0.041
**BMI***	26.57 (1.23)	26.87 (1.23)	26.31 (1.24)	0.108	0.099
**GCS on ED Presentation***	10.62 (1.81)	10.81 (1.8)	10.43 (1.83)	0.142	0.059
**ISS (Injury Severity Score)***	19.8 (1.6)	19.52 (1.6)	20.08 (1.6)	0.101	0.060
**Length of Hospital Stay (days)***	3.8 (3.83)	4.26 (3.4)	3.38 (4.24)	<0.001	0.172
**Length of ICU Stay (days)***	2.15 (3.08)	2.34 (2.92)	1.98 (3.24)	<0.001	0.150
**Total Hospital Charges***	46,051.76 (2.73)	47,165.17 (2.71)	44,964.63 (2.75)	0.193	0.048

* The values displayed are computed from the geometric mean
