# Supplementary material for: Racial and Ethnic Inequities in Mortality During Hospitalization for Traumatic Brain Injury: A Call to Action
Source: Front Surg. 2021 Jun 2;8:690971. doi: 10.3389/fsurg.2021.690971 (PMC8207515; doi:10.3389/fsurg.2021.690971)
Supplement: Supplementary file 2 [file Data_Sheet_2.pdf]

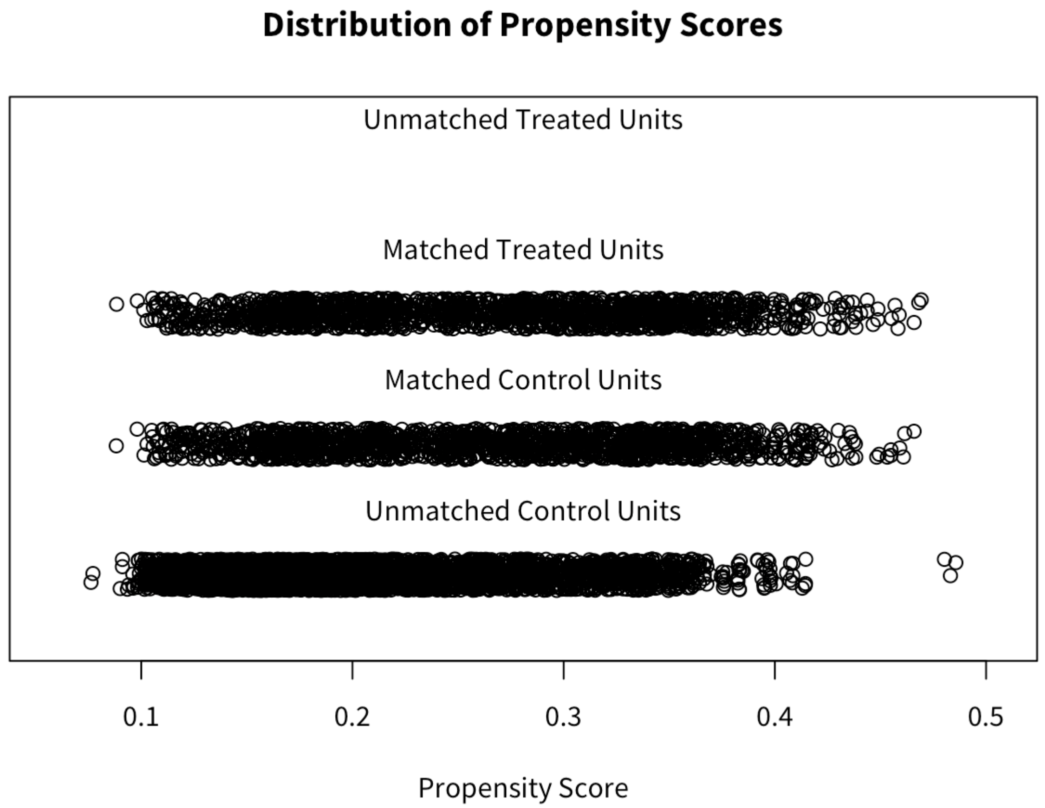

Supplementary Figure 1. Jitter plot of distribution of propensity scores before and after matching, including unmatched controls.

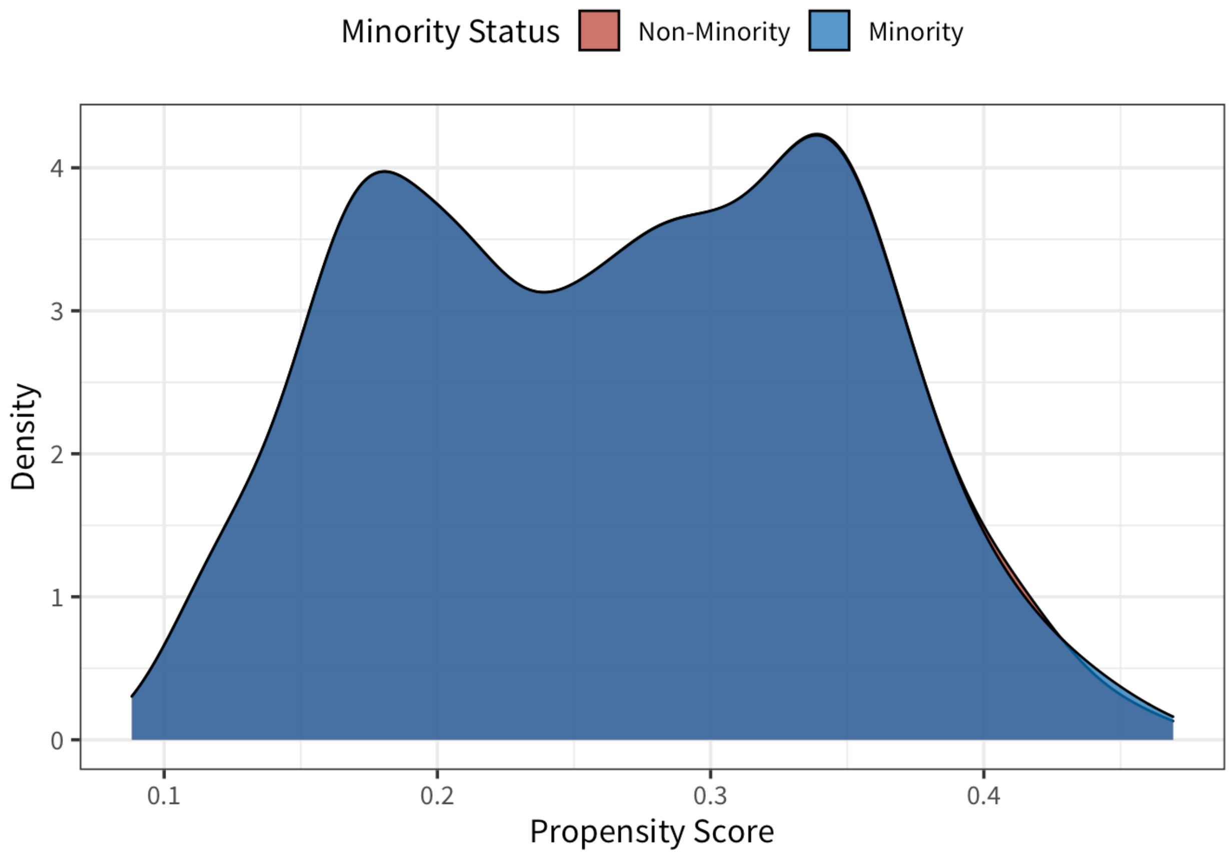

Supplementary Figure 2. Histogram of density of propensity score distribution demonstrating region of common support.

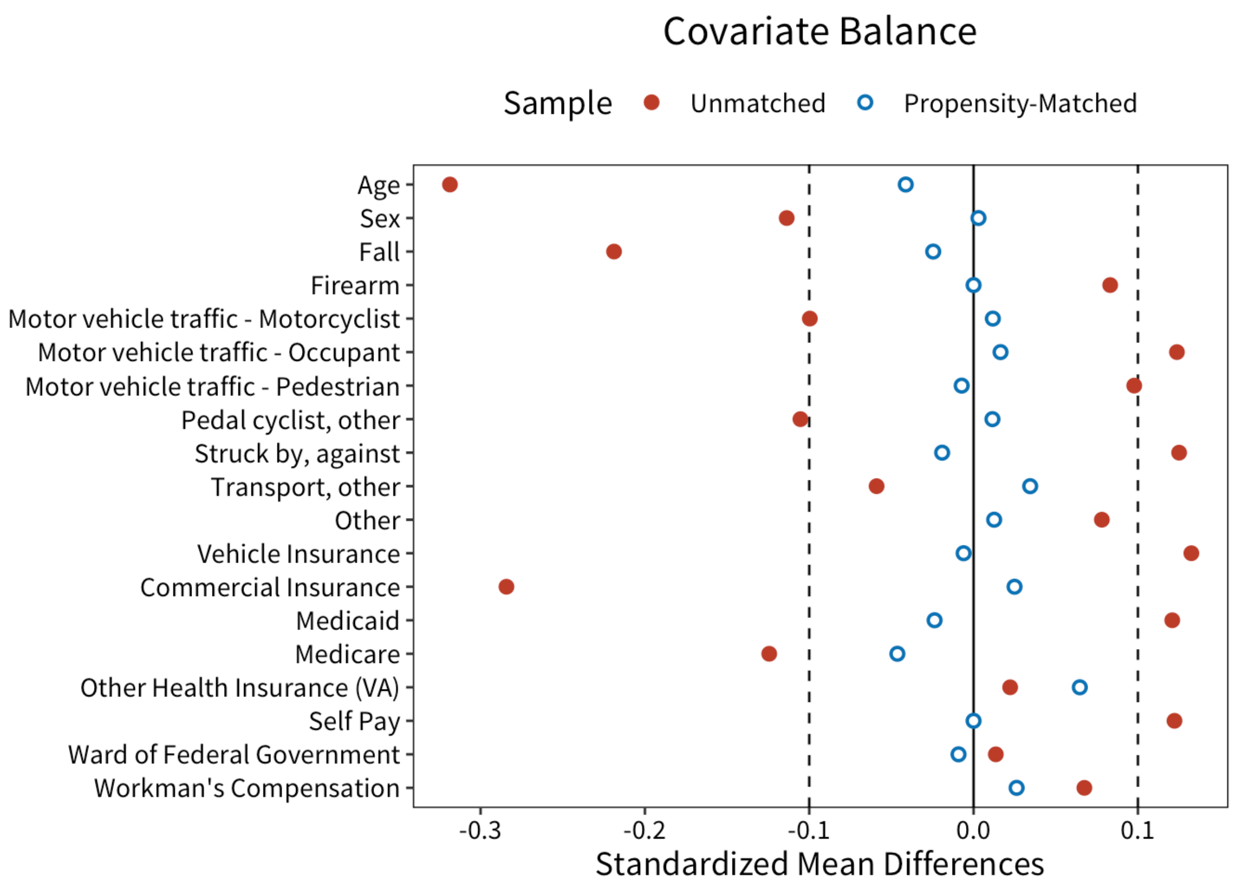

Supplementary Figure 3. Graphical comparison of standardized mean differences assessing covariate balance before and after propensity score matching.
